# How are central foveal and choroidal thickness affected in patients with mild coronavirus disease 2019 infection?

**DOI:** 10.17305/bjbms.2021.5840

**Published:** 2021-12

**Authors:** Müge Fırat, Sabiha Güngör Kobat

**Affiliations:** Department of Ophthalmology, Elazığ City Hospital, Elazığ, Turkey

**Keywords:** Central foveal thickness, choroidal thickness, coronavirus disease 2019, optical coherence tomography

## Abstract

The aim of this study was to evaluate the effects of coronavirus disease 2019 (COVID-19) on central foveal and choroidal thicknesses. Thirty-two patients with a positive severe acute respiratory syndrome coronavirus 2 polymerase chain reaction test who received outpatient treatment within the previous 2 months and 32 healthy controls were included in the study. Patients requiring hospitalization due to COVID-19 as well as the patients who received either intensive care support and/or antiplatelet therapy, smokers, or patients with systemic or ocular diseases were excluded from the study. After full ophthalmological examination, central foveal and choroidal thicknesses were evaluated using optical coherence tomography. Statistical analysis of the study data demonstrated no significant difference between the groups in terms of age or gender (p > 0.05). There was also no statistically significant difference between the groups in terms of central foveal thickness, central choroidal thickness, or nasal 500, nasal 1500, temporal 500, or temporal 500 micron distances (p > 0.05 for all parameters). Choroidal and retinal thicknesses were not affected in patients with recent mild COVID 19 without comorbidities.

## INTRODUCTION

Coronavirus disease 2019 (COVID-19) is a new disease caused by the severe acute respiratory syndrome coronavirus 2 (SARS-CoV-2) with high mortality rates affecting most frequently the airways and the lungs. Although it mainly affects the respiratory system, it can also affect cardiovascular, neurological, gastrointestinal, hepatic, renal, hematological, ocular, and cutaneous tissues [1]. Vascular lesions due to SARS-CoV-2 infection are caused by extensive thrombosis, microangiopathy, and angiogenesis which occur as a result of the severe damage to the vascular endothelium [2]. The virus uses angiotensin converting enzyme 2 (ACE 2) receptors to enter the cells [3]. Immunohistochemical analyses performed on human eyes have shown that ACE 2 receptors are found in the conjunctiva and corneal epithelium, ciliary body, choroid, retina, and retinal pigment epithelium (RPE) [4]. The virus is thought to cause endothelial damage by direct invasion of tissues, as well as microvascular damage and ischemia through the complement system [5]. Casagrande et al. investigated 14 individuals who were autopsied after death caused by COVID 19 and reported that SARS-Cov-2 ribonucleic acid was detected in the retina of three cases, thus proving the presence of the virus in ocular tissue [6].

The choroid is one of the most vascularized tissues in the human body which plays an important role in the oxygenation and nutrition of the outer retina, retinal temperature regulation, positional state of the retina, and secretion of growth factors [7]. In the light of these findings, it has been proposed that COVID-19 can affect the retina and choroid. Our extensive literature review revealed limited previous studies evaluating choroidal thickness in COVID-19 patients.

The purpose of this study was to evaluate the effects of COVID-19 on the macula and choroid.

## MATERIALS AND METHODS

Thirty-two patients diagnosed with COVID-19 within the previous 2 months, who have had completed a 14-day quarantine period (Group 1) and 32 healthy volunteers (Group 2), all aged 20–50, who presented to the Elazığ City Hospital Ophthalmology Outpatient Clinic, Turkey, for eye check-ups, were included in this study. Age- and gender-matched healthy individuals with no ocular or systemic diseases were enrolled in the control group (Group 2). Complete ophthalmological examinations were performed on all participants. Central foveal thickness and choroidal thickness in both groups were measured using optical coherence tomography (OCT).

### Inclusion criteria

Patients who were symptomatic for COVID-19 in the previous 2 months and had a positive reverse transcriptase-polymerase chain reaction (PCR) test result obtained from a nasopharyngeal swab, and who have had received favipiravir therapy for 5 days and recovered were included in the study.

### Exclusion criteria

Patients requiring hospitalization due to COVID-19 infection, who have received intensive care support and/or have received platelet antiplatelet therapy, smokers, or patients with systemic diseases, were excluded from the study. Patients with myopia, macula hole, cystoid macular edema, epiretinal membrane or vitreomacular traction documented by OCT, media opacities, vitreous hemorrhage, uveitis, choroidal neovascularization, and diabetic maculopathy/retinopathy, and patients with a history of previous intraocular interventions such as vitreoretinal, cataract or glaucoma surgery or intravitreal injection and macular laser photocoagulation as well as patients with low OCT image quality were excluded from the study.

### OCT scanning and analysis

**Figure 1:**
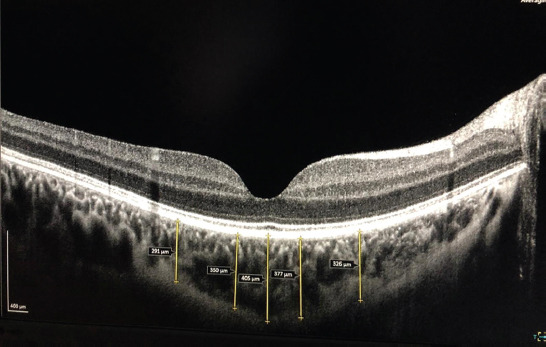
Choroidal thickness measurements on Canon optical coherence tomography (OCT)-HS 100 high resolution cross mode scans. Vertical lines are drawn from the posterior edge of the retinal pigment epithelium to the sclera using Canon OCT-HS 100 software.

Central foveal and the choroidal thicknesses were measured using OCT (Canon OCT-HS 100). The Canon OCT-HS100 device has a scanning speed of 70,000 A-scans/s with 3-mm axial resolution and a 2-mm scanning depth. The choroid mode, which gives a high-quality image, was used during the measurements. Choroidal thickness was measured vertically between the outer border of the hyperreflective line corresponding to the RPE and the inner surface of the sclera. Choroidal thickness was measured from the fovea and at 500 and 1500 mm nasally and temporally to the fovea (Figure 1). All measurements were performed by the same experienced blinded individual.

Assessments were made at 12:00 pm to avoid diurnal variations. Both eyes were evaluated in all patients in both groups. However, only the right eye measurements were included in the analysis.

### Statistical analysis

Statistical Package for the Social Sciences (SPSS) version 22.0 software (SPSS Inc., Chicago, IL, USA) was used for statistical analysis. The Kolmogorov–Smirnov test was applied to determine whether distribution between the groups was normal in terms of age, gender, central foveal thickness, and choroidal thickness. The independent sample *t*-test was used since distribution was normal in terms of age, gender and choroidal thickness, while the Mann–Whitney U-test was for central foveal thickness since distribution was not normal. *p* < 0.05 was considered statistically significant.

### Ethical statement

This observational, retrospective, comparative, and case–control study was conducted with the approval of the Fırat University Non-Invasive Research Ethics Committee (no. 2020/15-21) and was carried out in accordance with the principles of the Declaration of Helsinki.

## RESULTS

Thirty-two patients with histories of COVID-19 between 14 and 60 days, 16 male and 16 female, with a mean age of 31.62 ± 10.31 years (Group 1) and a healthy control group, 16 male and 16 female, with a mean age of 35.18 ± 7.27 years (Group 2) were included in the study. There was no significant difference between the groups in respect of age or gender (*p* = 0.11 and *p* = 1.00, respectively). Mean central foveal thickness was 242.71 ± 4.37 mm in Group 1 and 248.84 ± 5.23 mm in Group 2 (*p* = 0.43). The mean central choroidal thickness was 418.50 ±5 1.89 mm and 413.25 ± 48.22 mm in Group 1 and Group 2, respectively (*p* = 0.67). Measurements taken 500 and 1500 mm nasal and temporal to the fovea were 400.43 ± 59.82 mm, 366.50 ± 62.08 mm, 392.06 ± 64.09 mm, and 370.93 ± 63.02 mm, respectively, in Group 1, and 380.68 ± 48.46 mm, 346.09 ± 57.97 mm, 386.62 ± 55.22 mm, and 360.59 ± 46.25 mm in Group 2 (*p* = 0.15, *p* = 0.17, *p* = 0.71, and *p* = 0.45, respectively). There was no statistically significant difference between the groups in terms of central foveal or choroidal thicknesses (Table 1).

**TABLE 1:**
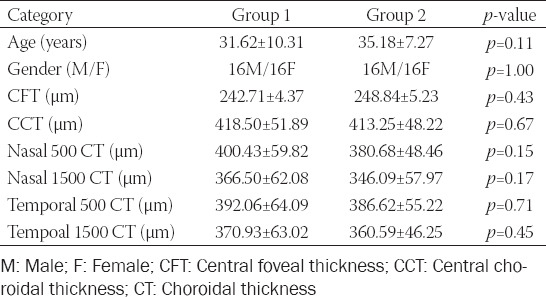
Age, gender, central foveal thickness, choroidal thickness, and p-value of the study groups

## DISCUSSION

The choroid is the layer of the eye with the highest vascularization and also the tissue with the highest blood flow per unit weight in the body [8]. It plays an important role in the physiology of the eye and pathogenesis of many ocular diseases. It also provides vascularization to the outer retina, the RPE, and some parts of the optic nerve [9]. It is also responsible for feeding the avascular fovea [10].

The choroid can be affected by a number of systemic viral infections, as well as chronic systemic diseases such as diabetes and hypertension. Thus far, studies have evaluated the relationship between the choroid and various viral infections, such as human immune deficiency virus and Epstein-Barr virus [11-14]. COVID-19 is known to affect the cardiovascular, neurological, gastrointestinal, hepatic, renal, hematological, ocular, and cutaneous tissues, but in particular it affects the respiratory system [1]. However, it is not yet fully understood which organs it affects, and how or what long-term consequences it may cause. Two possible mechanisms have been proposed for the systemic hematogenous pathway in COVID-19 infection ocular involvement – direct infection through microvascular endothelial cells expressing ACE2 and CD147, and/or spreading through infected leukocytes that can also cross the blood retinal barrier and carry the virus. Both processes can lead to retinal and choroidal infections [15].

Studies have also reported that COVID-19 infection affects different parts of the eye. Seah and Agrawal reported that COVID-19 can exhibit various ocular findings from anterior segment pathologies, such as conjunctivitis and anterior uveitis, to vision-threatening posterior segment pathologies, such as retinitis and optic neuritis [16]. Bettach et al. reported small focal intraretinal hemorrhage in the fovea and bilateral anterior uveitis in a PCR-negative and IgG-positive patient [17]. Hyper-reflective lesions in the inner plexiform layer and ganglion cell layers in the fundus at OCT examination between 11 and 33 days were reported in 12 patients with COVID-19 infection. Cotton wool spots and microhemorrhages in the retinal arcuate were observed in four patients. The authors speculated that the retinal findings in these patients might be due to acute inflammation [18]. In contrast, Vavvas et al. reported that these hyper-reflective spots seen at OCT reveal characteristic morphological and location features of the inner retinal vessels [19]. Paracentral acute middle maculopathy in one patient and acute macular neuroretinopathy in another were reported following COVID-19 infection [20]. Pereira et al. reported acute vascular lesions (hemorrhage and cotton wool spots) in patients with severe COVID-19 [21]. Retinal hemorrhages and cotton wool spots can also be seen in other systemic pathologies, and there were patients with comorbidities in this study. Therefore, it remains unclear whether those findings could have been attributed to these comorbidities.

Coagulopathies associated with thromboembolic events can also be seen in COVID-19 patients. A thrombotic complication rate of 31.7% has been reported in intensive care patients with COVID-19 [22]. Retinal artery occlusion has also been observed with COVID-19 [23]. Twenty-two percent of retinal microangiopathy is reported to be encountered at an average of 43 days after the first symptom of COVID-19 [24]. Papillophlebitis was also observed in a patient with a history of COVID-19. Inflammation and coagulopathy related to viral infection were described as the only risk factors in this patient with no systemic disease [25].

Simao et al. evaluated the retinas of COVID-19 patients who recovered between 20 and 83 days. They reported no significant infection-related change in the retina, optic disc, or retinal vascularity [26]. However, there were participants with various comorbidities in that study, and the severity of the disease was not the same in all participants. Zapata et al. divided patients who had contracted COVID-19 in the previous 3 months into three groups according to their clinical severity. OCT, fundus fluorescein angiography, and color fundus photographs of the patients were taken and evaluated. Decreased central retinal vascular density was observed in patients with moderate and severe COVID-19 [27]. Another study reported that retinal vein diameter correlated directly with the severity of the disease [28].

## CONCLUSION

The present study examined central foveal and choroidal thicknesses (nasal and temporal) using OCT in patients with COVID-19. Although there was a minimal decrease in central foveal and choroidal thicknesses in patients with COVID-19, this was not statistically significant (*p* > 0.05). We examined a period from 14 to 60 days after the onset of the first COVID-19 symptom, and also included patients who did not require intensive care or hospitalization, did not require anticoagulants, and did not have any chronic diseases. Thus, confounding that might have resulted from comorbidities and anticoagulant use with COVID-19 was eliminated. In addition, COVID-19 patients included in the study had received 5 days of favipiravir therapy. Our extensive review of the literature did not reveal any study of favipiravir-related ocular involvement. Since the duration of antiviral use was limited to 5 days in this study, no change due to antiviral use was expected.

This study has several limitations. First of all, OCT may not detect all of the choroidal vascular changes. Second, not all patients were seen during their early acute and untreated phase. Furthermore, not all patients who have experienced severe COVID-19 symptoms were seen during this phase. Thus, this study does not exclude possible choroidal changes that may have occurred during the early acute phase or in severe cases.

Examining patients with additional diseases or requiring intensive care hospitalization may have prevented objective results being obtained in the previous studies. Therefore, no patients with additional disease or using anticoagulants that might have affected choroidal thickness were included in this study. This made it possible to reveal the effects of COVID-19 in the fovea and choroid more clearly. In addition, evaluating a period commencing after 14 days after the onset of first symptoms enabled the exclusion of temporary changes that might occur due to acute inflammation. To the best of our knowledge, this study is unique from these perspectives.

This study shows that the infection has no short-term effect on nasal and temporal choroidal as well as on subfoveal retinal thicknesses in patients with a history of mild COVID-19 and without comorbidities.
